# Integrative Transcriptome and Metabolome Profiles Reveal Common and Unique Pathways Involved in Seed Initial Imbibition Under Artificial and Natural Salt Stresses During Germination of Halophyte Quinoa

**DOI:** 10.3389/fpls.2022.853326

**Published:** 2022-04-12

**Authors:** Huifang Yan, Yuting Nie, Kailun Cui, Juan Sun

**Affiliations:** Grassland Agri-Husbandry Research Center, College of Grassland Science, Qingdao Agricultural University, Qingdao, China

**Keywords:** salt stress, quinoa, seed initial imbibition, transcriptome, metabolome

## Abstract

Salt stress is a major environmental factor that seriously restricts quinoa seed germination. However, the key regulatory mechanisms underlying the effect of salt stress on the initial imbibition stage of quinoa seeds are unclear. In this study, dry seeds (0 h) and imbibed (8 h) seeds with 450 mM NaCl (artificial salt) and 100% brackish water of Yellow River Estuary (BW, natural salt) were used to assess the key salt responses based on germination, transcriptome, and metabolome analyses. The results indicated that the capacity of germinating seeds to withstand these two salt stresses was similar due to the similarities in the germination percentage, germination index, mean germination time, and germination phenotypes. Combined omics analyses revealed that the common and unique pathways were induced by NaCl and BW. Starch and sucrose metabolism were the only commonly enriched pathways in which the genes were significantly changed. Additionally, amino sugar and nucleotide sugar metabolism, and ascorbate and aldarate metabolism were preferably enriched in the NaCl group. However, glutathione metabolism tended to enrich in the BW group where *glutathione peroxidase*, *peroxiredoxin 6*, and *glutathione S-transferase* were significantly regulated. These findings suggest that the candidates involved in carbohydrate metabolism and antioxidant defense can regulate the salt responses of seed initial imbibition, which provide valuable insights into the molecular mechanisms underlying the effect of artificial and natural salt stresses.

## Introduction

Quinoa (*Chemopodium quinoa*), an annual herbaceous plant of the Chenopodiaceae family, is mainly cultivated for its edible seeds and leaves (Ruiz et al., 2016). Its gluten-free seeds contain various nutrients including fiber, minerals, vitamins, natural antioxidants, a high protein content (Gordillo-Bastidas et al., 2016), and large amounts of essential amino acids, such as lysine, that is usually limited in cereals (Panuccio et al., 2014). Moreover, quinoa has superior resistance to multiple abiotic stresses including cold, drought, and salt, and it even can grow under inclement environments that are unsuitable for many major crops (Park and Hahn, 2021). In addition, quinoa has the outstanding salt tolerance compared with other cereals, such as wheat (*Triticum aestivum*) and corn (*Zea mays*), other vegetables, and forages (Hinojosa et al., 2018). In recent years, quinoa has attracted much attention from scientists because of its important salt tolerance (Orsini et al., 2011), and several studies have assessed the response of quinoa leaves and roots to salt stress (Nguyen et al., 2020; Shi and Gu, 2020). Nevertheless, salinity significantly affects the seed germination stage during the whole life cycle of quinoa (Yang et al., 2018). Most studies that analyze the inhibitory effects of salinity on quinoa seed germination mainly focus on phenotypic traits (leaf area, root length, and root/shoot ratio) and biochemical determinations (antioxidant enzyme, soluble sugar, proline, protein, and inorganic ions) (Stoleru et al., 2019; Cai and Gao, 2020). To date, however, the comprehensive response mechanism involved in the seed germination stage under high salt stress at the transcriptomic and metabolomic levels are unknown.

Soil salinization mainly occurs in the coastal and semi-arid areas and restricts plant growth and development, thus reducing productivity (Turan et al., 2012). Soil salinity affects more than 800 million hectares of land globally, which may rapidly increase in the future by reason of global climate changes (Ilangumaran and Smith, 2017). In China, saline soils cover a vast area of the total arable land and coastal alluvial regions. Simultaneously, saline soils also experience alkalization, which is even more toxic to plants than single salinization (Feng et al., 2021). For instance, the Yellow River Estuary (YRE) (Shandong Province, China) is a densely populated saline-alkaline mudflat area with diversified cultures and economic activities. It is inescapably affected by saline-alkalization caused by continuous seawater intrusion and salinity changes (Zhao Q. et al., 2020). It is reported that quinoa can grow in various soils and areas with poor ecological environments in China, thus playing a vital role in saline-alkaline soil amelioration (Yang X. et al., 2019). Many previous studies mainly focused on the effects of artificial salt stress (NaCl, Na_2_SO_4_, and Na_2_CO_3_) on quinoa, with only a few analyzing the effect of natural salt stress (seawater, brackish water of the YRE). Therefore, it is necessary to study the effect of salt stress to uncover the related mechanism involved in seed germination under natural salt stress, which will contribute to providing a foundation for developing this halophyte as a pioneer crop for improving and utilizing saline soils.

Salt stress detrimentally affects almost all the major physiological processes of plants, including germination, morphogenesis, photosynthesis, and yield formation (Acosta-Motos et al., 2017). It is documented that highly saline concentrations limit plant life events through osmotic stress, ion toxicity, and secondary oxidative stress by reducing water absorption and inducing the accumulation of ions and reactive oxygen species (ROS) (Zhang Y. et al., 2019). However, plants have evolved a series of adaptive mechanisms in response to salt stress effects by regulating gene expression and metabolite synthesis (Pandey and Pandeyrai, 2016). Some key osmoprotectants, such as amino acids, organic acids, and sugars, are specifically synthesized under salt stress and function in regulating cellular osmotic potential (Wang et al., 2016). Previous findings in sesame (*Sesamum indicum*) have shown that strong salt-induced accumulation of sugars, including maltose, raffinose, sucrose, and trehalose, can protect membranes and proteins from degradation (Zhang Y. et al., 2019). Moreover, plants induce defense strategies to reduce lipid peroxidation triggered by excessive ROS production under salt stress via both enzymatic and non-enzymatic mechanisms to maintain the redox homeostasis (Demidchik, 2015). The pivotal enzymes involved in the ascorbate - glutathione (AsA-GSH) cycle, including ascorbate peroxidase (APX), glutathione peroxidase (GPX), and glutathione S-transferase (GST) (essential for ROS detoxification), alleviate the oxidative damage to macromolecules and cells (Nazar et al., 2015). Causin et al. (2020) reported that NaCl stress restricted seed germination of three genotypes of quinoa. However, it showed that the activity profile of antioxidant enzymes was not related to the salt tolerance of genotypes. To date, the responses of osmolytes and antioxidant enzymes of quinoa seeds to salt stress at the germination stage are unclear.

Salinity obviously inhibits seed germination, from imbibition to radicle protrusion through the seed coat (Zhang Y. et al., 2019). Seed germination consists of three phases based on water absorption: a rapid water uptake period (phase I), a plateau of water uptake (phase II), and a rapid increase of water uptake with growth (phase III) (Cheng et al., 2017). The initial imbibition stage (phase I) plays a vital role during seed germination by providing basis for reactivating the necessary metabolic and physiological processes involved in phase II (He et al., 2011). Studies have recently assessed seed germination characteristics of different quinoa cultivars or genotypes based on biometric measurements and biochemical indexes to quantify the tolerance to salinity (Stoleru et al., 2019; Causin et al., 2020). Additionally, the transcriptomic analyses have been used to partially uncover the differential gene expression profiles of quinoa in answering to salt stress (Shi and Gu, 2020), thus providing a preliminary understanding of the underlying regulation mechanism of seed germination. However, most genes related to quinoa seed germination are still unknown due to the complexity of the germination process. In addition, no study has comprehensively evaluated the molecular mechanisms and the relationship between transcriptomic and metabolomic changes of quinoa in response to salt stress during germination. Therefore, in this study, the transcriptome and metabolome profiles of quinoa seeds were integratively analyzed under artificial salt solution (450 mM NaCl, SC group) and natural salt solution (100% brackish water of the YRE, BW group) stresses to identify the common and differential metabolic pathways involved in initial imbibition stage during germination. This study will provide valuable insights into the molecular mechanisms underlying the salt tolerance of quinoa.

## Materials and Methods

### Seed Materials

Quinoa (cultivar “Longli No. 4”) seeds were obtained from Animal Husbandry, Pasture and Green Agriculture Institute (Lanzhou, Gansu Province, China). The seeds were harvested at maturity in 2019, with original germination percentage (GP) of 100% and moisture content of 11.58% (fresh weight basis). The plump and uniform seeds were selected and stored in −20°C refrigerator in darkness for further use.

### Seed Germination Under Salt Stress

Seed germination assay was performed as described by Panuccio et al. (2014). Three replicates of fifty seeds each were imbibed in a petri dish (110 × 110 mm) with three layers of filter paper wet with 10 ml of distilled water, NaCl solution (300, 450, and 600 mM), and BW solution (40%, 80%, and 100%, from YRE, 37°35′2″ N, 118°56′19″ E, Dongying, Shandong Province, China). Seeds were incubated in the dark at a constant temperature of 25°C for five days. The saline solutions were replaced every 12 h to maintain the NaCl and BW concentrations. Germination was defined as radicle protrusion (at least 2 mm) through the seed coat (Causin et al., 2020). The number of germinated seeds was recorded every 12 h. The GP, germination index (GI), and mean germination time (MGT) were then calculated (Abdul-Baki and Anderson, 1973). Based on the screening results, 450 mM NaCl and 100% BW were selected to further study the underlying mechanisms of salt stress on seed initial imbibition. The ion composition, concentration, pH, and conductivity values of saline solutions were also determined (Supplementary Table 1).

### Establishment of Seed Imbibition Curve Under Salt Stress

The original seed moisture content was assayed following the International Seed Testing Association (ISTA) rules (ISTA, 2019). A total of 100 seeds were weighed and imbibed in a petri dish under 10 ml of 450 mM NaCl or 100% BW in the dark at 25°C (three replicates). The weight of imbibed seeds was measured every 4 h to calculate the moisture content. The seed imbibition curve was then established to determine the germination phases.

### Seed Samples for Transcriptome and Metabolome Analyses

The 8 h (at phase I) was determined as the initial imbibition stage of germination based on the seed imbibition curve. Mature and plump quinoa seeds were imbibed under 450 mM NaCl or 100% BW in the dark at 25°C for 8 h. These were then immediately collected and frozen in liquid nitrogen and stored at −80°C for transcriptome and metabolome analyses. The dry seeds were used as control (CK). All treatments were carried out with three independent replicates.

### RNA Isolation, cDNA Library Construction, and Sequencing

Total RNA was extracted from CK, SC, and BW seed samples using Trizol reagent (Invitrogen, Carlsbad, CA, United States), of which the purity and integrity were assessed by strict quality control. Poly-T oligo-attached magnetic beads were used to purify mRNA obtained from total RNA. The cDNA double-stranded synthesis, purification, and adaptor ligation were conducted, then the fragments were enriched to construct the sequencing library. An Agilent Bioanalyzer 2100 system was used to assess the quality of the library. The constructed cDNA library was sequenced on an Illumina HiSeq™ 4000 platform (San Diego, CA, United States) to create 150 bp paired end reads.

### Quality Control, *de novo* Assembly, and Annotation

Raw data were filtered by removing the reads with adapter pollution. Afterwhich, more than 10% of unknown nucleotides (N), and more than 50% of low-quality (Q value ≤ 20) bases, and the remaining highly qualitative clean reads were then subjected to the downstream analyses. The hierarchal index for spliced alignment transcipts (HISAT; version 2.1.0) was used to map clean reads to the reference genome of quinoa (ASM168347v1, NCBI) (Kim et al., 2015). The Trinity program was used for *de novo* assembly of transcriptome with default parameter values (Grabherr et al., 2011). The functional annotations of the assembled unigenes were obtained from public databases using local BLAST programs (version 2.2.28) at a threshold e-value ≤ 1e^–5^. Gene Ontology (GO) annotation (including biological process, cellular component, and molecular function) of all unigenes was obtained using Blast2GO program (version 3.0.8) at a threshold e-value ≤ 1e^–5^. Meanwhile, Kyoto Encyclopedia of Genes and Genomes (KEGG) annotation was achieved using KEGG Automatic Annotation Server (KAAS) at a threshold e-value ≤ 1e^–10^.

### Identification and Enrichment Analysis of Differentially Expressed Genes

The expression level of genes was calculated using Fragments Per Kilobase of transcript per Million fragments mapped (FPKM) to evaluate the length and depth of sequencing. The DESeq2 software (version 1.22.1) was used for differential expression analysis of each gene between the pairwise comparison of CK, SC, and BW seed samples (Love et al., 2014). The corrected *p*-value generated with the Benjamini-Hochberg procedure was used to control the False Discovery Rate (FDR). The DEGs were identified based on FDR and fold change (FC) of genes between the three groups, with a level of | log_2_FC| ≥ 1 and FDR < 0.05 as the threshold. GO enrichment and KEGG analysis were performed using GO-seq R packages and KOBAS software, respectively, to predict the potential biological functions and pathways of DEGs (Mao et al., 2005; Young et al., 2010).

### Metabolome Profiling Analysis

For metabolite extraction, the CK, SC, and BW seed samples were prepared and sampled from the same batch as those used for transcriptome analysis. Each treatment had three biological replicates. The seeds (1 g) of each replicate were freeze-dried and crushed into fine powder. About 100 mg of lyophilized powder was dissolved with 1.2 ml of 70% methanol, vortexed after every 30 min (six times in total, each for 30 s), and then incubated at 4°C overnight. The solution sample was centrifuged at 12,000 rpm for 10 min. The supernatants were filtrated (SCAA-104, 0.22 μm pore size, ANPEL, Shanghai, China) before ultra-performance liquid chromatography tandem mass spectrometry (UPLC-MS/MS) analysis.

A UPLC-electrospray ionization (ESI)-MS/MS system (UPLC, SHIMADZU Nexera X2^1^; MS, Applied Biosystems 4500 Q TRAP^2^) was used to analyze the prepared sample extracts. The UPLC analysis system was equipped with a C18 column (Agilent SB-C18, 1.8 μm, 2.1 × 100 mm). The analysis was performed using a binary solvent system consisting of pure water (with 0.1% formic acid) as mobile phase A and acetonitrile (with 0.1% formic acid) as mobile phase B. Samples were measured using a gradient program that started with the A: B (v/v) of 95%: 5% at 0 min. A linear gradient to 5% A and 95% B was programmed within 9 min and kept for 1 min. Subsequently, a composition of 95% A and 5% B was adjusted within 1.1 min and maintained for another 2.9 min. The injection volume, flow velocity, and column temperature were 4 μl, 3.5 ml/min, and 40°C, respectively. The effluent was alternately connected to an ESI-triple quadrupole-linear ion trap (QTRAP)-MS.

Linear ion trap (LIT) and triple quadrupole (QQQ) scans were obtained using an AB4500 Q TRAP UPLC-MS/MS system which was equipped with an ESI Turbo Ion-Spray interface operating in positive and negative ion modes and controlled via Analyst 1.6.3 software (Applied Biosystems Company, Framingham, MA, United States). The operation parameters of ESI source were as follows: ion source, turbo spray; source temperature of 550°C; ion spray voltage (IS) of 5500 V in positive ion mode and −4500 V in negative ion mode; ion source gas I (GSI), gas II (GSII), and curtain gas (CUR) were 50, 60, and 25.0 psi, respectively; and the collision-activated dissociation (CAD) was set at high. Instrument tuning and mass calibration were performed using 10 and 100 μM polypropylene glycol solutions, respectively, in QQQ and LIT modes. The QQQ scans were acquired as MRM experiments with collision gas (nitrogen) set at medium. A specific set of MRM transitions were supervised for each period based on the metabolites eluted within this period.

Analyst 1.6.3 software was used to analyze the raw data of the metabolite spectrum. The principal component analysis (PCA) was performed using the statistics function of “prcomp” within R (version 3.5.0). The hierarchical cluster analysis (HCA) was carried out using the R package pheatmap (version 1.0.12). Differentially abundant metabolites (DAMs) were selected based on the combination of variable influence on projection (VIP) value extracted from the orthogonal projections to latent structures discriminant analysis (OPLS-DA) model and | log_2_FC| calculated from fold change of metabolites between the groups. Metabolites with VIP ≥ 1 and | log_2_FC| ≥ 1 were considered as DAMs. Identified metabolites were annotated using KEGG Compound database^3^. The KEGG pathway database^4^ was used to construct metabolite pathways.

### Conjoint Analysis of Transcript and Metabolite

To estimate the concordance between transcriptome and metabolome, the square of the Pearson Correlation Coefficient (R^2^) was calculated based on the fold change of DEGs and DAMs between the groups. Correlations corresponding to a coefficient with R^2^ > 0.8 and *p* < 0.05 were used as the threshold.

### Validation of Transcriptomic Data by QRT-PCR

To verify the reliability of transcriptomic data, ten DEGs involved in seed initial imbibition during salt stress were selected for qRT-PCR. The RNA was extracted using the Plant RNA Kit V1.5 (Biofit, Chengdu, China), and the cDNA synthesis was performed using a Goldenstar™ RT6 cDNA Synthesis Kit Ver2 (TSK302S, Tsingke, Beijing, China). The qRT-PCR reaction system contained 10 μl of 2 × T5 Fast qPCR Mix (SYBR Green I) (TSE202, Tsingke, Beijing, China), 0.8 μl of forward primer, 0.8 μl of reverse primer, 1 μl of cDNA, and 7.4 μl of ddH_2_O, reaching a final volume of 20 μl. The qRT-PCR analysis was carried out on a LineGene 9600 Plus instrument (FQD-96A, Bioer, Hangzhou, China) with the following program: 95°C for 1 min, followed by 40 cycles at 95°C for 15 s, 60°C for 15 s, and 72°C for 30 s. All samples were examined in three biological replicates with three technological replicates. The quinoa *Elongation Factor 1*α (*EF1*α) was used as the reference gene. The relative expression levels of the ten DEGs were calculated via the 2^–ΔΔCt^ method (Livak and Schmittgen, 2001). Primers of genes for qRT-PCR were designed using the Primer-BLAST of NCBI^5^ (Supplementary Table 2).

### Statistical Analysis

The data obtained from three replicates were expressed as means ± SE. All statistical analyses were performed using SPSS software (SPSS Inc., version 17.0). Duncan’s test or *t*-test was used to evaluate the difference among the treatments at 5% significant level. Graphics were drawn using SigmaPlot software (Systat Software, Inc., version 10.0).

## Results

### Germination Characteristics of Quinoa Seeds Under Salt Stress

**FIGURE 1:**
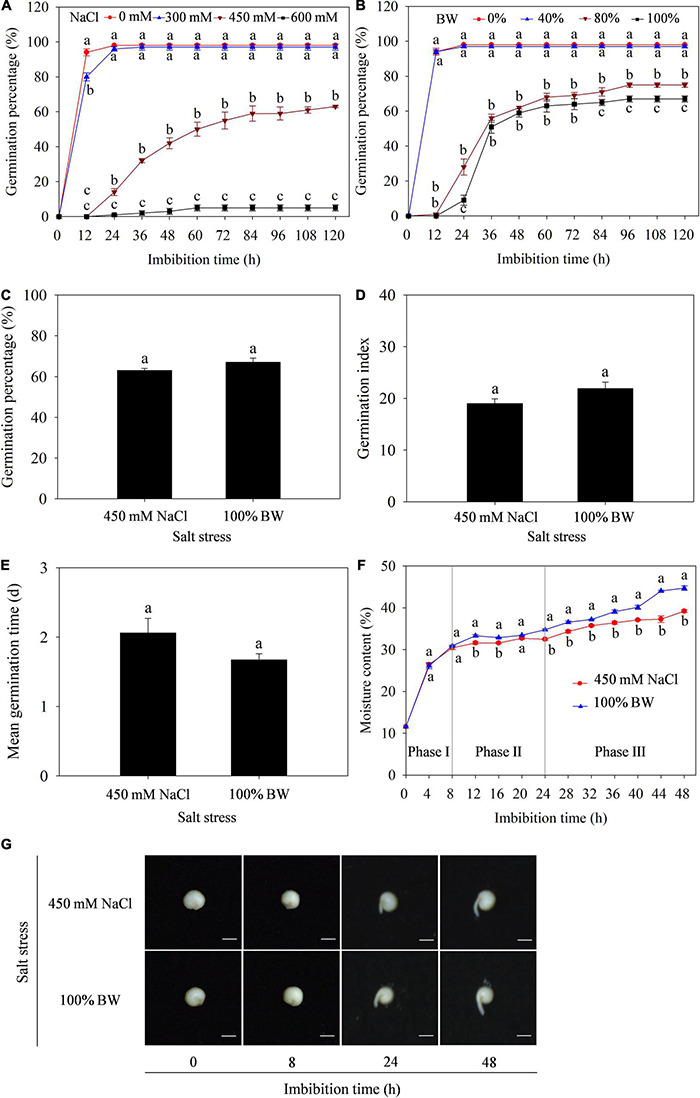
Effects of salt stress on germination of quinoa seeds. Germination percentage under different concentrations of **(A)** NaCl (0, 300, 450, and 600 mM) and **(B)** brackish water (BW; 0%, 40%, 80%, and 100%). Comparison of **(C)** germination percentage, **(D)** germination index, and **(E)** mean germination time under 450 mM NaCl and 100% BW stresses. **(F)** Dynamic changes of moisture content in imbibed seeds during germination under 450 mM NaCl and 100% BW stresses. **(G)** Seed germination phenotypes at three phases of imbibition under 450 mM NaCl and 100% BW. Different lowercase letters in **(A)**, **(B)**, and **(F)** indicated significant differences among different salt concentrations or salt stresses at the same imbibition time. Vertical bars represent standard error of three replicates. Means followed by different lowercase letters are significantly different at *p* < 0.05.

To determine the effect of salt stress on germination of quinoa seeds, the GP was primarily measured under different concentrations of NaCl (0, 300, 450, and 600 mM) and BW (0, 40, 80, and 100%). The results showed that GP was significantly affected at 450 and 600 mM NaCl (Figure 1A), and 80 and 100% BW (Figure 1B). Further analysis found that the capacity of germination to withstand salt stress was similar at 450 mM NaCl and 100% BW, since all GP, GI, and MGT were not different between the two salt stresses (Figures 1C-E). Additionally, to assess the initial imbibition stage of germination in quinoa, the dynamic changes of moisture content in imbibed seeds were analyzed during germination. The seed imbibition curve under the two salt stresses showed that the first 8 h of imbibition was related to a rapid increase of moisture content (Phase I), followed by a plateau of water uptake (Phase II) from 8 to 24 h, and a rapid increase of water uptake from 24 to 48 h (phase III, Figure 1F). Meanwhile, seed germination phenotypes at the three phases showed that the radicles did not protrude through the seed coat at Phase I (before 8 h). There were also no macroscopic germination differences between the two salt stresses (Figure 1G). Thus, the 8 h period (at phase I) was determined as the initial imbibition stage of germination. Transcriptome and metabolome analyses of the dry seeds (0 h) and seeds imbibed (8 h) with 450 mM NaCl (SC group) and 100% BW (BW group) were conducted to uncover gene and metabolite responses at seed initial imbibition stage of quinoa germination.

### Overview of Transcriptomic Responses of Seed Initial Imbibition Under Salt Stress

To explore the gene expression events of seed initial imbibition under salt stress, the imbibed seeds treated with or without 450 mM NaCl and 100% BW at 8 h underwent transcriptome analysis. A total of 42,820,532, 43,565,764, and 43,499,874 clean reads, 43,547,808, 42,278,838, and 46,028,320 clean reads, and 44,637,796, 43,977,552, and 46,557,854 clean reads were obtained from three biologically repeated samples of CK, SC, and BW groups, respectively (Supplementary Table 3). The proportions of Q20 and Q30 were 97.38-97.88% and 92.80-93.81%, respectively, and the GC content of clean reads was 42.85-44.02% (Supplementary Table 3), indicating that the transcriptome sequencing data were of high quality. The clean reads were mapped in the reference genome sequences of quinoa with the mapping ratio of 93.48-96.86% and the uniquely mapped reads of 88.89-93.03% (Supplementary Table 3). Moreover, a total of 44,001 unigenes were functionally annotated based on GO, KEGG, Eukaryotic Orthologous Groups of Protein (KOG, clusters of orthologous groups of proteins), non-redundant protein sequences (NR, NCBI non-redundant protein sequences), SwissProt, TrEMBL, and protein family (Pfam) databases, in which 30,564, 22,628, 20,454, 20,739, 27,404, 24,546, and 32,903 unigenes were annotated, respectively (Supplementary Table 4).

**FIGURE 2:**
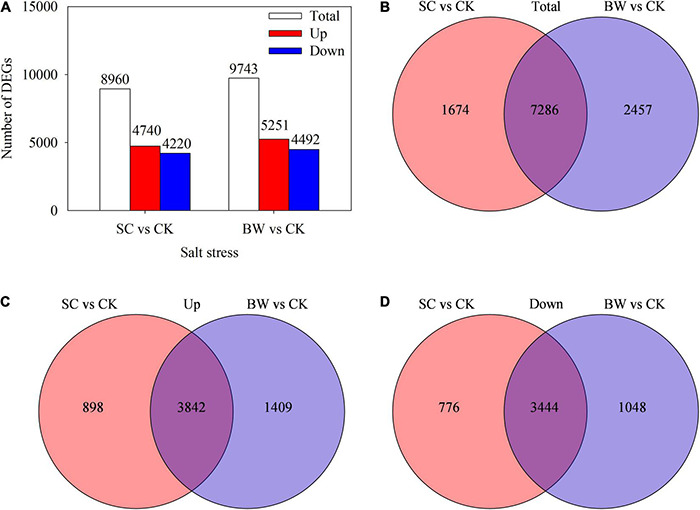
Profiles of differentially expressed genes (DEGs) involved in the seed initial imbibition under salt stress. **(A)** Number of the total upregulated and downregulated DEGs with fold change ≥ 2. Venn diagram of **(B)** total, **(C)** upregulated and **(D)** downregulated DEGs.

Based on the gene differential expression threshold of | log_2_FC| ≥ 1 and FDR < 0.05, a total of 8,960 (4,740 upregulated and 4,220 downregulated) and 9,743 (5,251 upregulated and 4,492 downregulated) DEGs were identified in the SC vs. CK and BW vs. CK comparisons, respectively (Figure 2A and Supplementary Tables 5, 6). Totally, 7,286 DEGs (3,842 upregulated and 3,444 downregulated) were commonly identified in both SC vs. CK and BW vs. CK (Figures 2B-D). Additionally, 1,674 (898 upregulated and 776 downregulated) and 2,457 (1,409 upregulated and 1,048 downregulated) DEGs were specifically changed in SC vs. CK and BW vs. CK, respectively (Figures 2B-D). These results indicated that both SC and BW could induce gene expression changes at the seed initial imbibition stage of germination in quinoa under salt stress. However, there were some differences between SC and BW regulations.

### The Functional Analysis of DEGs Involved in Seed Initial Imbibition Under Salt Stress

**FIGURE 3:**
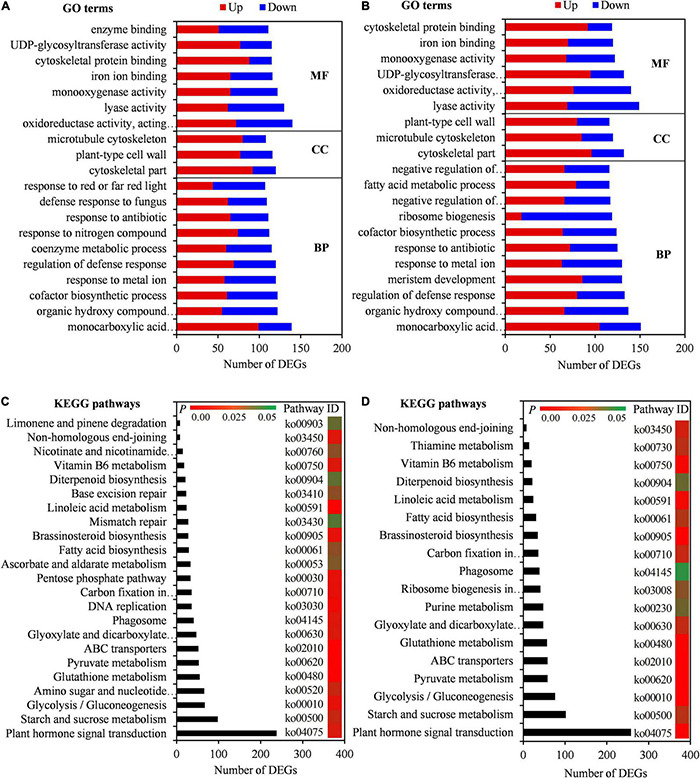
DEGs enriched on Gene Ontology (GO) terms and Kyoto Encyclopedia of Genes and Genomes (KEGG) pathways under NaCl (SC) and BW stresses. Top 20 GO terms of DEGs in **(A)** SC vs. control (CK) and **(B)** BW vs. CK. BP, CC, and MF represent biological process, cellular component, and molecular function, respectively. Significantly enriched KEGG pathways of DEGs in **(C)** SC vs. CK and **(D)** BW vs. CK.

To confirm the biological functions of seed initial imbibition under salt stress, the DEGs were subjected to GO analysis. A total of 3,985 GO terms were enriched in SC vs. CK, of which 2,614, 437, and 934 were involved in the biological process, cellular component, and molecular function, respectively. Similarly, 3,979 GO terms were enriched in BW vs. CK, of which 2,603, 436, and 940 were involved in the biological process, cellular component, and molecular function, respectively (Supplementary Table 7). The top 20 GO terms, including organic hydroxy compound metabolic process, cofactor biosynthetic process, regulation of defense response, cytoskeletal part, plant-type cell wall, oxidoreductase activity, monooxygenase activity, and cytoskeletal protein binding, showed that DEGs in both SC vs. CK and BW vs. CK were involved in salt tolerance in plants (Figures 3A,B). Moreover, the DEGs in SC vs. CK were highly enriched in several GO terms, including coenzyme metabolic process, response to nitrogen compound, and defense response to fungus (Figure 3A). However, the DEGs in BW vs. CK were highly enriched in GO terms of meristem development, ribosome biogenesis, negative regulation of biosynthetic process, fatty acid metabolic process, and negative regulation of cellular biosynthetic process (Figure 3B). These results indicated that there were partially different responses of seed initial imbibition under SC and BW salt stresses.

To further determine the molecular mechanisms of seed initial imbibition under salt stress, KEGG enrichment analysis of DEGs was conducted. A total of 131 KEGG pathways were enriched in either DEGs in SC vs. CK or BW vs. CK. Interestingly, they were all common under the two salt stresses (Supplementary Table 7). In this study, the significantly enriched KEGG pathways were mainly focused on due to their key roles under salt stress. The DEGs in SC vs. CK and BW vs. CK were involved in 23 and 18 similar pathways, respectively, including plant hormone signal transduction, starch and sucrose metabolism, glycolysis/gluconeogenesis, glutathione metabolism, pyruvate metabolism, ABC transporters, and fatty acid biosynthesis (Figures 3C,D). Additionally, the DEGs in SC vs. CK were also significantly enriched in pathways of amino sugar and nucleotide sugar metabolism, DNA replication, and ascorbate and aldarate metabolism (Figure 3C). However, the DEGs in BW vs. CK were also involved in pathways of purine metabolism and ribosome biogenesis (Figure 3D). These results suggested that although the underlying mechanisms of seed initial imbibition under SC and BW stresses were similar, the roles of the several exclusive pathways were different.

### Metabolomics Analysis of Seed Initial Imbibition Under Salt Stress

**FIGURE 4:**
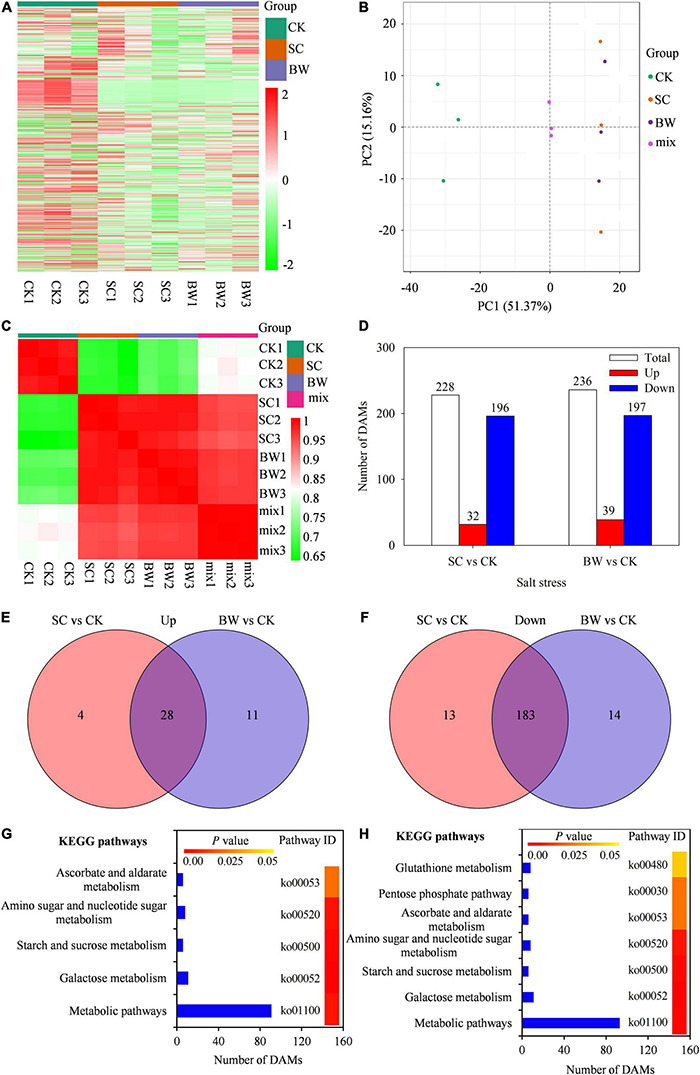
The metabolites analysis in quinoa during the seed initial imbibition under salt stress. **(A)** Heatmap visualization of total metabolites, **(B)** Principal Component Analysis (PCA) clustering based on metabolome data, **(C)** the correlation of samples based on the square of Pearson Correlation Coefficient (R^2^), **(D)** number of total, up-accumulated and down-accumulated differentially abundant metabolites (DAMs) with fold change ≥ 2, venn diagram of **(E)** up-accumulated and **(F)** down-accumulated DAMs, significantly enriched KEGG pathways of DAMs in **(G)** SC vs. CK and **(H)** BW vs. CK.

To further study the metabolite accumulation of seed initial imbibition under salt stress, the widely targeted metabolome analysis was performed using UPLC-ESI-MS/MS system. In total, 700 metabolites were detected in all samples (Figure 4A and Supplementary Table 8). The PCA was then performed using three quality control samples (mix) and nine samples. Results showed that the mix samples were located in the center, indicating that the data quality of the metabolome was good (Figure 4B). Meanwhile, the PCA revealed a clear separation between the control and salt stress by PC1 (Figure 4B). Additionally, the square of Pearson Correlation Coefficient (R^2^) analysis showed that the three biological replicates had a good correlation under the same salt stress (Figure 4C). Based on a threshold of | log_2_FC| ≥ 1, a total of 228 (32 up-accumulated and 196 down-accumulated) and 236 (39 up-accumulated and 197 down-accumulated) DAMs were detected in SC vs. CK and BW vs. CK, respectively (Figure 4D and Supplementary Table 9). Among these DAMs, there were 28 up-accumulated and 183 down-accumulated ones commonly detected in both SC vs. CK and BW vs. CK groups. In addition, the number of up-accumulated DAMs specifically changed in SC vs. CK and BW vs. CK were four and 11, respectively (Figure 4E), while the down-accumulated DAMs were 13 and 14, respectively (Figure 4F). The DAMs were summarized into 254 species which were mainly classified into lipids (65), organic acids (28), phenolic acids (28), amino acids and derivatives (25), nucleotides and derivatives (21), alkaloids (19), flavonoids (14), lignans and coumarins (5), terpenoids (5), steroids (1), and others (Supplementary Table 9).

The KEGG classification of DAMs showed that five and seven KEGG pathways were significantly enriched in SC vs. CK and BW vs. CK, respectively, suggesting that these metabolic pathways might play significant roles during seed initial imbibition under salt stress (Figures 4G,H). However, DAMs in SC vs. CK and BW vs. CK were significantly enriched in metabolic pathways, galactose metabolism, starch and sucrose metabolism, amino sugar and nucleotide sugar metabolism, and ascorbate and aldarate metabolism. Furthermore, DAMs in BW vs. CK were also significantly enriched in the pathways of glutathione metabolism and pentose phosphate pathway (Figure 4H).

### The Conjoint Analysis of Transcriptomics and Metabolomics Changes of Seed Initial Imbibition Under Salt Stress

To determine the relationship between DEGs and DAMs at seed initial imbibition stage under salt stress, the conjoint analysis of transcriptomics and metabolomics data was performed, with R^2^ > 0.8 (or < −0.8) and *p* < 0.05. The conjoint analysis showed that the starch and sucrose metabolism pathway was simultaneously connected by transcriptome and metabolome in both SC vs. CK and BW vs. CK (Supplementary Table 10). In addition, the conjoint analysis also revealed that amino sugar and nucleotide sugar metabolism and ascorbate and aldarate metabolism were significantly enriched only in SC vs. CK. In contrast, glutathione metabolism was significantly enriched only in BW vs. CK (Supplementary Table 10). These results suggested that salt stress could regulate the co-expression of DEGs and DAMs associated with amino sugar and nucleotide sugar metabolism, starch and sucrose metabolism, ascorbate and aldarate metabolism, and glutathione metabolism of seed initial imbibition during germination in quinoa. Thus, the above pathways were further analyzed below.

### The Integrative Analysis of Genes and Metabolites Involved in Starch and Sucrose Metabolism of Seed Initial Imbibition Under Salt Stress

**FIGURE 5:**
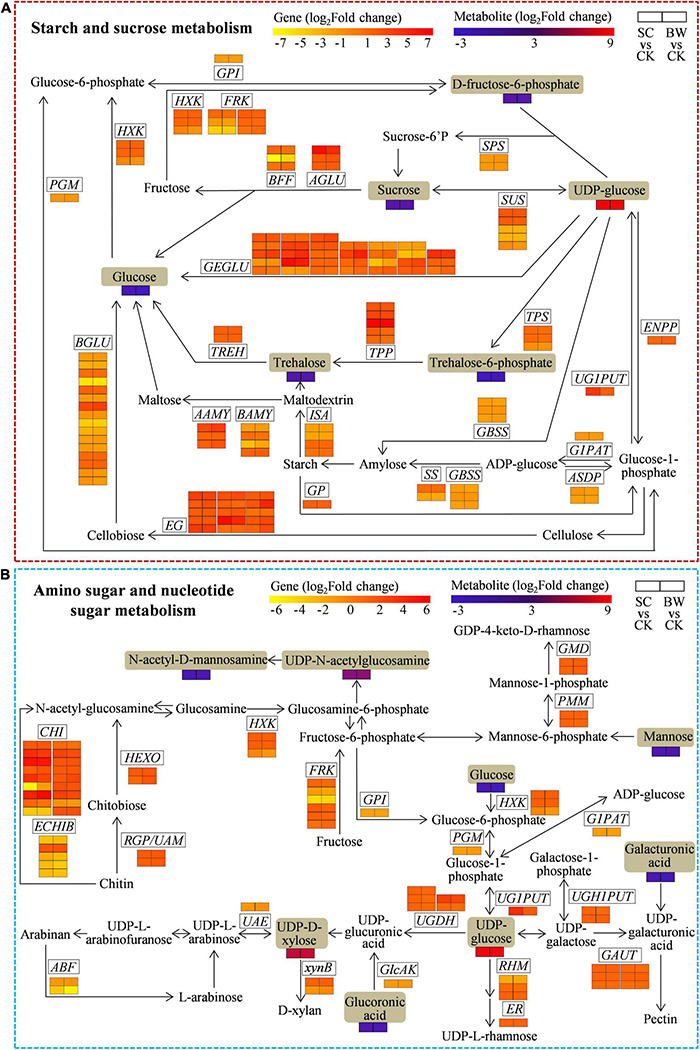
The DEGs and DAMs involved in **(A)** starch and sucrose metabolism and **(B)** amino sugar and nucleotide sugar metabolism during the seed initial imbibition under salt stress. The brown background indicated the DAMs that changed under salt stress. The rectangular color block was equally divided into two parts, of which the left represented DEGs or DAMs in SC vs. CK, and the right represented DEGs or DAMs in BW vs. CK.

To assess the genes and metabolites related to starch and sucrose metabolism of seed initial imbibition under salt stress, the interaction between DEGs and DAMs was analyzed (Figure 5A and Supplementary Table 11). Notably, this pathway was simultaneously connected by the two omics in both SC vs. CK and BW vs. CK where 98 (63 upregulated and 35 downregulated) and 102 (65 upregulated and 37 downregulated) DEGs were identified, respectively (Supplementary Tables 10, 11). Although most DEGs involved in starch and sucrose metabolism were upregulated under both SC and BW stresses, genes regulating some enzymes were downregulated. For instance, *UTP-glucose-1-phosphate uridylyltransferase* (*UG1PUT*, LOC110681807), *trehalose 6-phosphate phosphatase* (*TPP*, LOC110709634), *endoglucanase* (*EG*, LOC110700982, LOC110722603, and LOC110736362), *alpha-glucosidase* (*AGLU*, LOC110711717), and *glucan endo-1,3-beta-glucosidase* (*GEGLU*, LOC110725108, LOC110728559, LOC110730004, and LOC110707358) were upregulated by 14.563, 118.578, 11.363, 13.730, 86.219, 79.625, 11.268, 12.882, 81.411, and 20.708-fold in SC vs. CK, respectively. Moreover, *TPP* (LOC110709634), *alpha-amylase* (*AAMY*, LOC110718929), *EG* (LOC110700982, LOC110736362, and LOC110701034), *beta-glucosidase* (*BGLU*, LOC110722071), and *GEGLU* (LOC110710484, LOC110725108, LOC110728559, LOC110720904, and LOC110707358) were upregulated by 104.001, 12.283, 10.073, 29.679, 32.302, 12.527, 13.311, 11.896, 24.450, 36.052, and 17.009-fold in BW vs. CK, respectively. The metabolites were significantly changed under both SC and BW stresses, especially the uridine 5′-diphospho-D-glucose (UDP-glucose) which was significantly accumulated by 314.414 and 359.676-fold, respectively (Supplementary Table 11). However, the other metabolites, such as sucrose, trehalose, trehalose 6-phosphate, fructose 6-phosphate, and glucose, were decreased. These results indicated that the DEGs and DAMs related to starch and sucrose metabolism conjointly acted in response to SC and BW stresses.

### The Integrative Analysis of Genes and Metabolites Involved in Amino Sugar and Nucleotide Sugar Metabolism of Seed Initial Imbibition Under Salt Stress

To explore the genes and metabolites related to amino sugar and nucleotide sugar metabolism of seed initial imbibition under salt stress, the interaction between DEGs and DAMs was analyzed (Figure 5B and Supplementary Table 12). This pathway was connected by both transcriptome and metabolome only in SC vs. CK, where 66 DEGs (48 upregulated and 18 downregulated) were identified (Supplementary Tables 10, 12). For instance, 5 *UDP-glucose 6-dehydrogenase* (*UGDH*), 2 *hexokinase* (*HXK*), 3 *fructokinase* (*FRK*), *UG1PUT*, 2 *UDP-glucose-hexose-1-phosphate uridylyltransferase* (*UGH1PUT*), 14 *chitinase* (*CHI*), 2 *GDP-mannose 4,6-dehydratase* (*GMD*), *hexosaminidase* (*HEXO*), *3,5-epimerase/4-reductase* (*ER*), 2 *reversibly glycosylated polypeptide/UDP-arabinopyranose mutase* (*RGP/UAM*), 5 *alpha-1,4-galacturonosyltransferase* (*GAUT*), and 2 *phosphomannomutase* (*PMM*) were upregulated, while *glucose-1-phosphate adenylyltransferase* (*G1PAT*), *alpha-L-arabinofuranosidase* (*ABF*), *glucose-6-phosphate isomerase* (*GPI*), *phosphoglucomutase* (*PGM*), *UDP-glucuronate 4-epimerase* (*UGA4E*), *UDP-arabinose 4-epimerase* (*UAE*), *glucuronokinase* (*GlcAK*), and 4 *basic endochitinase B* (*ECHIB*) were downregulated. Notably, *UG1PUT* (LOC110681807) and *CHI* (LOC110716695, LOC110716931, and LOC110718981) were upregulated by 14.563, 35.679, 13.296, and 21.586-fold, respectively. Moreover, the metabolites were significantly changed in SC vs. CK, of which uridine 5’-diphospho-N-acetylglucosamine (UDP-GlcNAc), UDP-glucose, and uridine-5’-diphosphate-D-xylose (UDP-D-Xyl) were significantly accumulated. Notably, UDP-glucose and UDP-D-Xyl were significantly increased by 314.414 and 43.239-fold, respectively. However, the other metabolites, including glucoronic acid, N-acetyl-D-mannosamine, mannose, and glucose, were all decreased. These results indicated that the DEGs and DAMs related to amino sugar and nucleotide sugar metabolism acted conjointly in response to SC stress.

### The Integrative Analysis of Genes and Metabolites Involved in Ascorbate and Aldarate Metabolism of Seed Initial Imbibition Under Salt Stress

**FIGURE 6:**
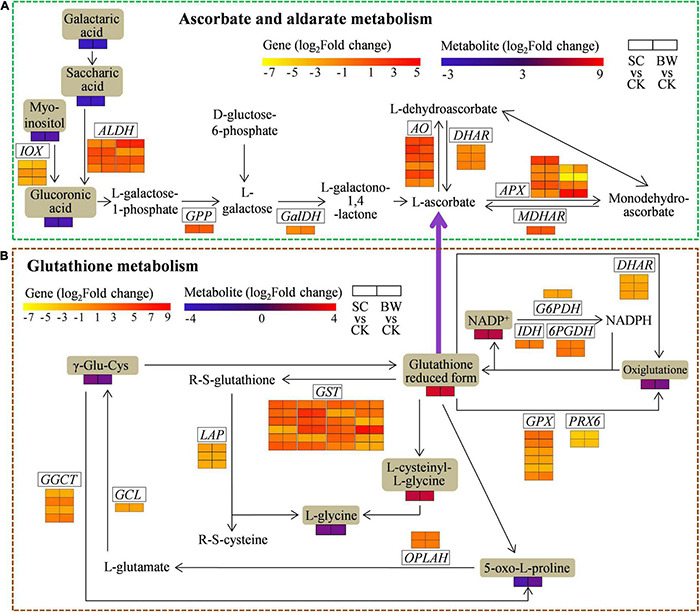
The DEGs and DAMs involved in **(A)** ascorbate and aldarate metabolism and **(B)** glutathione metabolism during the seed initial imbibition under salt stress. The brown background indicated the DAMs that changed under salt stress. The rectangular color block was equally divided into two parts, of which the left represented DEGs or DAMs in SC vs. CK and the right represented DEGs or DAMs in BW vs. CK.

To assess the genes and metabolites related to ascorbate and aldarate metabolism of seed initial imbibition under salt stress, the interaction between DEGs and DAMs was analyzed (Figure 6A and Supplementary Table 13). This pathway was also only connected by both transcriptome and metabolome in SC vs. CK, where 33 DEGs (17 upregulated and 16 downregulated) were detected (Supplementary Tables 10, 13). For example, 4 *aldehyde dehydrogenase (NAD* +) (*ALDH*), 4 *L-ascorbate oxidase* (*AO*), and *monodehydroascorbate reductase (NADH)* (*MDHAR*) were upregulated, while 3 *ALDH*, 5 *APX*, 3 *inositol oxygenas*e (*IOX*), *L-galactose dehydrogenase* (*GalDH*), and 2 *glutathione dehydrogenase/transferase* (*DHAR*) were downregulated. It should be noted that *ALDH* (LOC110694134) and *AO* (LOC110717796) were upregulated by 8.936 and 7.210-fold, respectively. Additionally, the metabolites were significantly changed in SC vs. CK. These above results indicated that the DEGs and DAMs related to ascorbate and aldarate metabolism acted conjointly in response to SC stress.

### The Integrative Analysis of Genes and Metabolites Involved in Glutathione Metabolism of Seed Initial Imbibition Under Salt Stress

To determine the genes and metabolites related to glutathione metabolism of seed initial imbibition under salt stress, the interaction between DEGs and DAMs was analyzed (Figure 6B and Supplementary Table 14). This pathway was only connected by both transcriptome and metabolome in BW vs. CK, where 57 (34 upregulated and 23 downregulated) DEGs were identified (Supplementary Tables 10, 14). Notably, *APX* (LOC110701632), *GST* (LOC110702644, LOC110724460, LOC110726460, LOC110727188, LOC110727191, and LOC110696395), and *ribonucleoside-diphosphate reductase subunit M2* (*RRM2*, LOC110681929 and LOC110695094) were upregulated by more than 10-fold. Notably, the *GST* (LOC110696395) was significantly upregulated by 119.716-fold, suggesting that *GST* might play a major role during seed initial imbibition under BW stress. Additionally, the downstream metabolites associated with glutathione metabolism, the glutathione reduced form and L-cysteinyl-L-glycine (CYS-GLY), were accumulated in BW vs. CK. These results indicated that the DEGs and DAMs related to glutathione metabolism acted conjointly in response to BW stress.

### The Quantitative Real-Time PCR (QRT-PCR) Validation of DEGs in Seed Initial Imbibition During Salt Stress

To evaluate the accuracy of transcriptome data, ten DEGs (*CHI*, *TPP*, *EG*, *ALDH*, *GST*, and *RRM2*) were selected from the starch and sucrose metabolism, amino sugar and nucleotide sugar metabolism, ascorbate and aldarate metabolism, and glutathione metabolism for qRT-PCR analysis. The trend of the relative expression level of ten DEGs between qRT-PCR and transcriptome data was consistent (Supplementary Figure 1). Interestingly, both qRT-PCR test and transcriptome sequencing showed that the ten DEGs had consistent upregulation under salt stress, which verified the credibility of transcriptome data.

## Discussion

Salt stress severely limits plant growth, development, and productivity (Turan et al., 2012). Moreover, salt stress significantly affects seed germination, the most important and sensitive initial stage of plant life events (Kaya et al., 2003). Therefore, it is necessary to determine the key responses and related mechanisms involved in seed initial imbibition of germination under salt stress. In this study, the transcriptomic and metabolomic changes in 8 h-imbibed quinoa seeds under SC and BW were studied, which will provide new insights into the candidate genes involved in seed initial imbibition of quinoa under salt stress.

### Hidden Differences of the Underlying Mechanisms Under the Similar Germination Characteristics in Response to Two Salt Stresses

Germination results showed that both SC and BW significantly decreased the GP and GI of quinoa seeds under salt stress, while lengthened the MGT. There was no difference in the germination parameters under SC and BW, indicating that the capacity of germination to withstand these two salt stresses was similar (Figure 1). Based on the determination of physicochemical properties of SC and BW, it showed that although the conductivity values of these two salt solutions were almost the same, BW had a lower ion concentration and a higher pH value than SC (Supplementary Table 1), demonstrating that the effect of BW on seed germination of quinoa was simultaneously affected by salt stress and alkaline stress. Alkaline stress generally induces the same stress factors as salt stress, such as osmotic stress, oxidative stress, and ion stress, which destroy the ion homeostasis and ROS balance (Fan et al., 2021). Alkaline stress has greater harm than salt stress, especially when combined with high-pH stress (Wang et al., 2008). Furthermore, previous studies have reported that high pH can affect the accumulation and balance of organic acids (Zhang et al., 2013).

Plant responses to salt stress are regulated in a complex manner which involves a large number of genes (Wang et al., 2019). Therefore, in this study, although the germination characteristics of quinoa seeds under SC and BW stresses were very similar, there might be differences in metabolic pathways and mechanisms of seed germination under these two salt stresses since that BW property was not exactly same as SC property, and it could induce higher pH due to the HCO_3_^–^ and CO_3_^2–^. To further validate this speculation, the transcriptome and metabolome profile data were conjointly analyzed, and results showed that both common and unique pathways were involved in seed germination of quinoa under these two stresses. For instance, the starch and sucrose metabolism pathway were simultaneously connected by DEGs and DAMs under both SC and BW stresses. Additionally, amino sugar and nucleotide sugar metabolism and ascorbate and aldarate metabolism pathways were only connected by DEGs and DAMs under SC stress. Glutathione metabolism pathway was only connected by DEGs and DAMs under BW stress.

### Starch and Sucrose Metabolism of Seed Initial Imbibition in Response to Salt Stress

Carbohydrate metabolism is the key response to abiotic stress in plants. Many sugars, such as sucrose, glucose, fructose, trehalose, maltose, and starch, are emerging as crucial molecules and substrates in mediating various metabolic reactions (Xu et al., 2021). These sugars provide an important energy basis for seed germination, plant growth, and development. Meanwhile, they can also regulate osmotic balance and remove excess ROS to maintain cell homeostasis under stress conditions (Krasensky and Jonak, 2012). In this study, functional enrichment analysis of DEGs revealed that many genes associated with starch and sucrose metabolism were induced at similar levels under SC and BW stresses (Figure 5A and Supplementary Table 11), indicating the crucial roles of sugars in regulating quinoa seed germination responses to salt stress. Sucrose is the dominant sugar reserve in cells. It consists of glucose and fructose, which helps plants to answer abiotic stress (Nemati et al., 2018). Sucrose synthase (SUS) and sucrose phosphate synthase (SPS) are two key enzymes involved in sucrose metabolism by catalyzing the production of UDP-glucose and sucrose-6-phosphate, respectively (Chen et al., 2018). Alpha-glucosidase (AGLU), as a carbohydrate digestive enzyme, catalyzes the hydrolysis of alpha-glucopyranoside bonds in oligosaccharides and disaccharides to yield glucose. Meanwhile, beta-fructofuranosidase (BFF) could also catalyze the conversion of sucrose to produce fructose and glucose which are further phosphorylated by hexokinase (HXK) or fructokinase (FRK) during glycolysis. This provides sufficient substrates for multiple metabolic pathways (Granot et al., 2014). Herein, transcriptomic data revealed that *SUS*, *AGLU*, *BFF*, *HXK*, and *FRK* were partially induced under SC and BW stresses, suggesting that sucrose metabolism could be activated in quinoa seed during germination under salt stress.

Starch, a simple molecule composed of glucose residues, is the most abundant storage for carbohydrate in plants (Cui et al., 2019). Various enzymes, including G1PAT, starch synthase (SS), and granule bound starch synthase (GBSS), control starch synthesis. Previous studies showed that most genes involved in starch biosynthesis were upregulated under salt stress (You et al., 2019). Additionally, activation of starch degradation under salt stress is suitable for sugar accumulation (Thalmann et al., 2016). Isoamylase (ISA) and amylase (AMY) are two important enzymes that specifically degrade starch to synthesize maltose. The beta-amylase (BAMY) is involved in starch degradation during seed germination. It is also related to resistance to abiotic restrictions (Chen et al., 2019). In this study, transcriptome analysis showed that several DEGs (*G1PAT*, *SS*, and *GBSS*) governing starch synthesis were downregulated. Moreover, most DEG encoding enzymes associated with starch degradation, including *AAMY*, *BAMY*, and *ISA*, were upregulated under salt stress (Figure 5A and Supplementary Table 11). These findings indicated that high salt stress could repress starch synthesis and promote starch expenditure, thus enhancing the production of more accessible maltose for seed germination.

Besides being a source of energy, sufficient trehalose also provides osmotic protection and stabilizes biomolecules by preventing denaturing during dehydration (Fernandez et al., 2010). Trehalose 6-phosphate synthase (TPS) is a rate-limiting enzyme that catalyzes the first step of trehalose biosynthesis from UDP-glucose to trehalose 6-phosphate, which is finally converted into soluble disaccharide trehalose through trehalose-6-phosphate phosphatase (TPP) catalysis (Lyu et al., 2013; Krasensky et al., 2014). Subsequently, alpha, alpha-trehalase (TREH) can specifically hydrolyze trehalose into glucose (Krasensky and Jonak, 2012). Xu et al. (2021) reported that NaCl stress induced the upregulation of *TPP* in oat (*Avena sativa*), resulting in an elevated level of trehalose. Consistent with previous findings, this research showed that trehalose synthesis-related DEGs, such as *TPS*, *TPP* and *TREH*, were upregulated under SC and BW stresses (Figure 5A and Supplementary Table 11), which was theoretically contributed to providing more energy for seeds to better adapt to salt stress during the germination process. Additionally, glucose can be generated from UDP-glucose through GEGLU catalysis. Furthermore, cellulases, including endoglucanase (EG), cellobiohydrolase, and β-glucosidase (BGLU), are essential for hydrolyzing cellulose into glucose via synergistic action (Gao et al., 2019). In this study, most *GEGLU* and *EG* genes were upregulated, while *BGLU* genes were downregulated, manifesting that these genes might play vital roles in trehalose metabolism during quinoa seed germination under salt stress.

Moreover, transcriptome data revealed that most DEGs involved in starch and sucrose metabolism under SC and BW were upregulated, suggesting that saccharide metabolism might be activated at seed initial imbibition stage of germination under salt stress. Nevertheless, combined with metabolome detection, it was found that the levels of various soluble sugars decreased, such as sucrose, glucose, trehalose, D-fructose-6-phosphate, and trehalose-6-phosphate derived from biosynthesis or starch degradation. Thus, these results showed that upregulation or downregulation of genes did not finally cause elevation or reduction of metabolites since various factors affect the process of gene expression to metabolites (Xu et al., 2021). Perhaps, in the early stage of seed germination under salt stress, the upregulated DEGs involved in starch and sucrose metabolism also increased sugars levels. However, the 450 mM NaCl was indeed a high level of salt stress, under which seeds needed to consume a large quantity of energy to maintain cell activities for germination. Therefore, the inconsistency in gene expressions and specific metabolite levels might be due to that salt stress activated DEGs associated with saccharide metabolism to synthesize more available sugars which were then rapidly transformed or consumed to meet the energy needs of seed germination under high salt stress. The decreased sugar levels further weakened the ability to maintain osmotic balance in cells which might also, on the other hand, aggravate the germination inhibition of quinoa seeds under salt stress.

### Amino Sugar and Nucleotide Sugar Metabolism of Seed Initial Imbibition in Response to Salt Stress

Glucose metabolism is of great importance for cell activities since it provides energy for plant events. Glucose is converted into glucose-6-phosphate, glucose-1-phosphate, and UDP-glucose via the catalysis of HXK, PGM, and UG1PUT that play crucial roles in polysaccharide biosynthesis, respectively (Ji et al., 2015). Notably, UDP-glucose, acting as the important branch node of converting UDP sugar, can be converted to UDP-glucuronic acid, UDP-D-xylose, and UDP-L-arabinose via UGDH and UAE catalysis through serial reactions (Han et al., 2020). Meanwhile, UDP-glucose can also be converted to UDP-galactose and UDP-galacturonic acid via GAUT catalysis. It can also be converted to UDP-L-rhamnose under UDP-glucose 4,6-dehydratase (RHM) and ER catalysis (Wang et al., 2017). Subsequently, these UDP sugar intermediates are further synthesized into L-arabinose, D-xylan, glucoronic acid, and pectin. Additionally, it is well known that the cell wall is a complex network structure that consists of cellulose, hemicellulose, and pectin (Liu et al., 2019). Multiple monosaccharides, including mannose, arabinose, rhamnose, and xylose, are involved in cell wall polysaccharides (Huang and Hou, 2021). Herein, most DEGs related to the synthesis of UDP sugars and downstream monosaccharide products were upregulated under SC stress, suggesting that nucleotide sugar metabolism might be activated at the initial imbibition stage of quinoa seed germination under salt stress (Figure 5B and Supplementary Table 12). Previous reports showed that cell wall construction, degradation, and modification played crucial roles in seed germination (Zhao J. et al., 2020). Particularly, the modification of pectins involved in the cell wall might limit of abiotic stress damage to cells due to forming hydrated gels (Leucci et al., 2008). The DEGs controlling enzymes associated with synthesizing UDP sugars and monosaccharides enhance response to imbibition related stresses at initial stage of seed germination. However, the relationship between these DEGs and cell wall function requires to be further investigated in quinoa.

Chitinase catalyzes the hydrolysis of chitin. Chitin is a major component of fungal cell wall, but it is not found in the plant cell wall (Grover, 2012; Xu et al., 2016). Plants have developed an immune system against invading fungi. Previous studies have shown that plant chitinase can hydrolyze the chitin in fungal cell walls or N-acetyl-glucosamine-containing glycoproteins in plant cell walls to release oligosaccharides, activating the immune responses of plants (Cao et al., 2019). Chitinase is also involved in plant response against abiotic stresses, such as osmotic, salt, cold, and heavy metal (Grover, 2012). In this study, transcriptomic data revealed that 14 *CHI* were upregulated under SC stress, indicating that the gene might be involved in response to salt stress at the initial imbibition stage of quinoa seed germination by producing oligosaccharides.

In summary, transcriptome data revealed that most DEGs related to amino sugar and nucleotide sugar metabolism were upregulated under SC stress. However, metabolome analysis showed that only UDP-D-xylose and UDP-glucose were significantly accumulated under SC stress. Other monosaccharides, such as glucose, mannose, and arabinose did not change under SC stress, indicating that UDP sugars might play positive roles in trying to offset the detrimental effects of SC stress on seed initial imbibition of germination in quinoa.

### Ascorbate and Aldarate Metabolism of Seed Initial Imbibition in Response to Salt Stress

Salt stress induces electron leakage of oxygen transport chain, resulting in the accumulation of harmful ROS and oxidative stress (Gao et al., 2019). Excess ROS causes seed germination inhibition and seedling growth delay (Yan et al., 2020). As a result, cells have evolved a complex ROS defense system for resistance against ROS attack (Ozgur et al., 2013). Ascorbate (AsA) is a strong and active antioxidant that can provide crucial protection against excessive ROS induced by salt stress (Zhang et al., 2014). Plants have two biosynthetic pathways of AsA. One pathway is from myo-inositol to L-galactose, where IOX catalyzes the first step to form glucuronic acid and L-galactose 1-phosphate phosphatase (GPP) catalyzes the intermediate reaction to form L-galactose. Meanwhile, glucuronic acid can also be formed from saccharic acid via ALDH catalysis. The other pathway is from D-gluctose-6-phosphate to L-galactose, which is subsequently oxidized by the key enzyme GalDH to form L-galactono-1,4-lactone, the direct precursor of AsA (Smirnoff et al., 2001). Herein, *IOX*, *ALDH*, *GPP*, and *GalDH* genes were transcriptionally regulated under SC stress (Figure 6A and Supplementary Table 13), suggesting that salt stress can affect AsA biosynthesis. Additionally, AO and APX are two major oxidases that catalyze AsA to form dehydroascorbate (DHA) or monodehydroascorbate (MDHA), which could be further converted to AsA via DHAR and MDHAR catalysis (Huang and Hou, 2021). In this study, the upregulation of *AO* and *MDHAR* and downregulation of *DHAR* and *APX* indicated that AsA was more likely to be transformed into a more stable DHA than MDHA during ROS scavenging process under salt stress (Figure 6A and Supplementary Table 13). However, the expression changes of metabolites and genes were not completely consistent, possibly due to other complex factors involved in seed initial imbibition under SC stress.

### Glutathione Metabolism of Seed Initial Imbibition in Response to Salt Stress

Glutathione reduced form (GSH), a tripeptide (γ-glu-cys-gly) and the major endogenous antioxidant involved in AsA-GSH cycle, acts as a substrate to quench ROS and eliminates damaging peroxides (Asthir et al., 2020). Two ATP-dependent enzymes catalyze GSH synthesis. Glutamate-cysteine ligase (GCL) catalyzes cysteine and glutamate to produce γ-glutamylcysteine (γ-Glu-Cys) which is subsequently converted to GSH via glutathione synthetase catalysis (Asthir et al., 2020). Meanwhile, GSH can be degraded via several different enzymes, of which gamma-glutamylcyclotransferase (GGCT) is the major one. GGCT hydrolyzes GSH to release 5-oxo-L-proline which is then converted to L-glutamate by 5-oxoprolinase (OPLAH) (Ohkama-Ohtsu et al., 2008). Herein, several genes involved in GSH synthesis and degradation, including *GCL*, *GGCT*, and *OPLAH*, were regulated under BW stress (Figure 6B and Supplementary Table 14), demonstrating that salt stress can affect GSH metabolism.

Nicotinamide adenine dinucleotide phosphate (NADPH) is a key cofactor, and its regeneration is essential in maintaining cellular redox homeostasis to defense against oxidative stress (Leterrier et al., 2012). Several NADPH-generating dehydrogenases, including isocitrate dehydrogenase (IDH), glucose-6-phosphate 1-dehydrogenase (G6PDH), and 6-phosphogluconate dehydrogenase (6PGDH), are involved in this process. In this study, one *IDH* and two *6PGDH* were upregulated under BW stress, comprehensively considering the accumulation of the NADP^+^ substrate (Figure 6B and Supplementary Table 14), indicating that NADPH generation might play a key role in resistance to salt stress.

Moreover, GPX and peroxiredoxin (PRX) are two antioxidant enzymes that play key roles in catalyzing the reduction of H_2_O_2_ by oxidizing GSH to form oxiglutatione (GSSG) (Zhang L. P. et al., 2019). Previous studies have shown that *GPX* overexpression in transgenic plants can induce tolerance to abiotic stresses, such as drought and salt (Diao et al., 2014; Kim et al., 2014). Besides, DHAR catalyzes DHA to form AsA in AsA-GSH cycle using GSH as the reducing substrate. In this study, three *GPX*, two *PRX6*, and two *DHAR* were downregulated together with the accumulated levels of GSH and L-cysteinyl-L-glycine (Figure 6B and Supplementary Table 14), indicating that GSH could be maintained at a higher level at the initial imbibition of germination under salt stress. GSTs are a group of multifunctional protective enzymes that utilize GSH to produce R-S-glutathione and are involved in plant defense responses by detoxifying ROS induced by abiotic stresses (Yang Q. et al., 2019). Xu et al. (2015) reported that overexpression of *AtGSTU19* in *Arabidopsis* promoted the resistance to salt stress by enhancing GST activity and maintenance of ROS balance. Herein, 22 *GST* genes (18 upregulated) involved in glutathione metabolism were identified under BW stress. Therefore, the expression of *GST* might contribute to eliminate ROS and minimize the damage induced by salt stress.

## Conclusion

**FIGURE 7:**
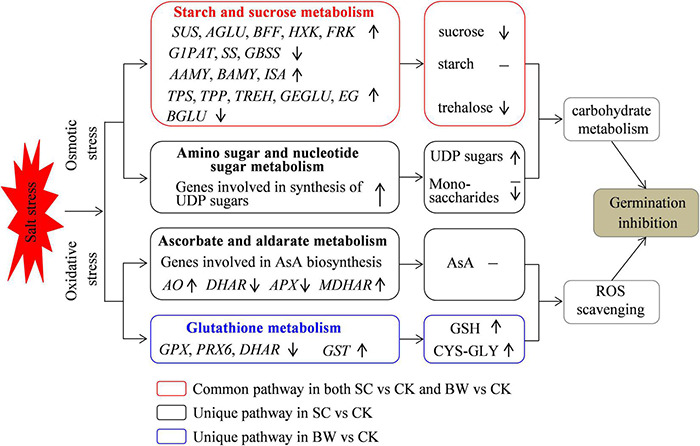
A proposed model for germination inhibition at the seed initial imbibition stage under salt stress. Osmotic stress and oxidative stress were induced and some related DEGs were regulated under SC and BW stresses during seed germination.

In summary, this study explored the possible mechanisms and key biological pathways involved in the initial imbibition stage of quinoa seed germination under SC and BW stresses (Figure 7). The integrative transcriptome and metabolome analysis successfully identified the regulatory candidates involved in starch and sucrose metabolism, amino sugar and nucleotide sugar metabolism, ascorbate and aldarate metabolism, and glutathione metabolism. The results suggested that osmotic stress and oxidative stress were induced, and some common and unique biological pathways were related to seed initial imbibition under these two salt stresses. Overall, this study confirmed that the major strategy of seed germination against salt stress was to regulate the carbohydrate metabolism and antioxidant defense function.

## Data Availability Statement

The datasets presented in this study can be found in online repositories. The names of the repository/repositories and accession number(s) can be found below: NCBI SRA BioProject with accession number of PRJNA795723.

## Author Contributions

HY, YN, and JS conceived and designed the experiments. HY, YN, and KC performed the experiments and analyzed the data. HY drafted and revised the manuscript. All authors have read and agreed to the version of the manuscript to be published.

## Conflict of Interest

The authors declare that the research was conducted in the absence of any commercial or financial relationships that could be construed as a potential conflict of interest.

## Publisher’s Note

All claims expressed in this article are solely those of the authors and do not necessarily represent those of their affiliated organizations, or those of the publisher, the editors and the reviewers. Any product that may be evaluated in this article, or claim that may be made by its manufacturer, is not guaranteed or endorsed by the publisher.

## Footnotes

1
www.shimadzu.com.cn/

2
www.appliedbiosystems.com.cn/

3
http://www.kegg.jp/kegg/compound/

4
http://www.kegg.jp/kegg/pathway.html

5
https://www.ncbi.nlm.nih.gov/tools/primer-blast/

## Supplementary Material

Supplementary Figure 1 The qRT-PCR validation of 10 DEGs.

Supplementary Table 1 The main ion composition, concentration, pH, and conductivityvalues of 450 mM NaCl and 100% BW.

Supplementary Table 2 The primer sequences of genes for qRT-PCR in this study.

Supplementary Table 3 The information of transcriptome.

Supplementary Table 4 The expression and annotation of total genes.

Supplementary Table 5 The expression and annotation of DEGs in SC vs. CK.

Supplementary Table 6 The expression and annotation of DEGs in BW vs. CK.

Supplementary Table 7 Information of GO and KEGG analysis of DEGs in SC vs. CK andBW vs. CK.

Supplementary Table 8 The information of metabolome.

Supplementary Table 9 The information of DAMs in SC vs. CK and BW vs. CK.

Supplementary Table 10 The KEGG pathways significantly enriched in at least one omics.

Supplementary Table 11 DEGs and DAMs involved in starch and sucrose metabolism.

Supplementary Table 12 DEGs and DAMs involved in amino sugar and nucleotide sugar metabolism.

Supplementary Table 13 DEGs and DAMs involved in ascorbate and aldarate metabolism.

Supplementary Table 14 DEGs and DAMs involved in glutathione metabolism.
