# Supplementary material for: Integrative Transcriptome and Metabolome Profiles Reveal Common and Unique Pathways Involved in Seed Initial Imbibition Under Artificial and Natural Salt Stresses During Germination of Halophyte Quinoa
Source: Front Plant Sci. 2022 Apr 12;13:853326. doi: 10.3389/fpls.2022.853326 (PMC9039654; doi:10.3389/fpls.2022.853326)
Supplement: Supplementary Figure 1 — The qRT-PCR validation of 10 DEGs. [file Image_1.docx]

**Supplementary Figure 1.** The qRT-PCR validation of 10 DEGs. *CHI*, *chitinase*; *TPP*, *trehalose 6-phosphate phosphatase*; *EG*, *endoglucanase*; *ALDH*, *aldehyde dehydrogenase (NAD+)*; *GST*, *glutathione S-transferase*; *RRM2*, *ribonucleoside-diphosphate reductase subunit M2*.
