# Supplementary material for: Integrative Transcriptome and Metabolome Profiles Reveal Common and Unique Pathways Involved in Seed Initial Imbibition Under Artificial and Natural Salt Stresses During Germination of Halophyte Quinoa
Source: Front Plant Sci. 2022 Apr 12;13:853326. doi: 10.3389/fpls.2022.853326 (PMC9039654; doi:10.3389/fpls.2022.853326)
Supplement: Supplementary Table 1 — The main ion composition, concentration, pH, and conductivityvalues of 450 mM NaCl and 100% BW. [file Table_1.docx]

**Supplemental Table 1.** The main ion composition, concentration, pH, and conductivity values of 450 mM NaCl and 100% BW.

| Items | Unit | 450 mM NaCl | 100% BW |
| --- | --- | --- | --- |
| Na^+^ | mM | 450 | 313.816 |
| K^+^ | mM | / | 7.926 |
| Fe (Fe^2+^+Fe^3+^) | mM | / | 0.00377 |
| Mn^2+^ | mM | / | 0.00345 |
| Cu^2+^ | mM | / | 0.00002 |
| Zn^2+^ | mM | / | 0.00004 |
| Ca^2+^ | mM | / | 9.726 |
| Mg^2+^ | mM | / | 19.544 |
| Cl^-^ | mM | 450 | 396.139 |
| SO_4_^2-^ | mM | / | 10.65 |
| HCO_3_^-^ | mM | / | 3.169 |
| CO_3_^2-^ | mM | / | 0.095 |
| pH | / | 6.230 | 7.904 |
| Conductivity | dS/m | 58.270 | 57.235 |

Note: BW, brackish water in the Yellow River Estuary (YRE).
